# Antimicrobial and Larvicidal Activities of Different Ocimum Essential Oils Extracted by Ultrasound-Assisted Hydrodistillation

**DOI:** 10.3390/molecules27051456

**Published:** 2022-02-22

**Authors:** Kuzhimbattil Sneha, Arunaksharan Narayanankutty, Joice Tom Job, Opeyemi Joshua Olatunji, Ahmed Alfarhan, Ademola C. Famurewa, Varsha Ramesh

**Affiliations:** 1 Division of Cell and Molecular Biology, PG and Research Department of Zoology, St. Joseph’s College (Autonomous), Devagiri, Calicut 673 008, Kerala, India; sneha1999@gmail.com (K.S.); joicetomj@gmail.com (J.T.J.); 2 Traditional Thai Medical Research and Innovation Center, Faculty of Traditional Thai Medicine, Prince of Songkla University, Hat Yai, Songkhla 90110, Thailand; 3 Department of Botany and Microbiology, College of Science, King Saud University, P.O. Box 2455, Riyadh 11451, Saudi Arabia; alfarhan@ksu.edu.sa; 4 Department of Medical Biochemistry, Faculty of Basic Medical Sciences, College of Medicine, Alex-Ekwueme Federal University, Ndufu-Alike Ikwo, P.M.B. 1010, Abakaliki 482131, Nigeria; ademola.famurewa@funai.edu.ng; 5 Department of Biotechnology, Deakin University, Geelong, VIC 3217, Australia; v.ramesh@deakin.edu.au

**Keywords:** *Ocimum essential oil*, Ultrasound Assisted Extraction, larvicidal property, antibacterial activity, antioxidant activity

## Abstract

Infectious diseases and their vectors have remained a concern for human population from their historical origin. Microbial pathogens have also emerged as a potent threat to the healthcare systems even in developed countries. Essential oils remain a less explored method for infectious disease control; besides, the ultrasound-assisted extraction (UAE) of essential oil production has emerged as promising source of bioactive volatiles over conventional methods. This study analyzed the possible use of UAE- Essential oils (EOs) from different species of Ocimum plants (*Ocimum basilicum* (OB), *O. gratissimum* (OG), *O. tenuiflorum* (OT), and *O. canum* (OC)) in the management of microbial pathogens and mosquito larval control. The antibacterial activity was estimated in terms of a disc diffusion assay and minimum inhibitory concentrations against *Escherichia coli, Staphylococcus aureus, Pseudomonas aeruginosa,* and *Salmonella enteritidis*. The larvicidal property was found using three important mosquito vectors and the LC_50_ value was determined. Furthermore, antioxidant and anti-inflammatory properties were estimated in terms of radical scavenging activities and the inhibition of lipoxygenase enzyme activity. The EOs exhibited significant DPPH radical scavenging (high in OG), hydrogen-peroxide scavenging (OB) and lipoxygenase inhibition (OB). The antibacterial activity was high in OB and OG (*p* < 0.05) and the larvicidal activity was of higher sensitivity against *Aedis* and *Culex*, whereas *Armigeres* was more resistant. However, no sign of toxicity in the *Allium cepa* model or non-targeted organism Guppy fishes was observed. Overall, the UAE extracted *Ocimum* essential oils were found to be effective against various human pathogenic microbial organisms, with OB and OG being highly active. Likewise, the EOs was also able to induce mortality in the larval forms of various mosquito vectors.

## 1. Introduction

Infectious diseases accounts for the greatest number of human and livestock death over the recent years [1]. Increased mortality has been associated with some of these diseases, including Ebola, influenza, pneumonia, and sepsis. Among the various pathogenic organisms, the microbial pathogens, especially bacterial diseases are the most important and are known to be associated with the highest mortality [2]. Additionally, the management cost of these microbial diseases is also high with respect to the medical expenses and sanitization expenses [3]. Furthermore, the antibiotic resistance that is emerging in various microbial pathogens enhances the risk of microbial disease-mediated health risks [4]. This has led to the search for novel antimicrobial agents, for which, the essential oils derived from aromatic plants are important drug candidates. 

Basil plants (*Ocimum* sp.) are widely used in various traditional medicinal systems including Ayurveda and Chinese medicines as well as folk medicines. The plants have also been important in various cultural and religious aspects in various countries. The plant has been accredited with innumerable health benefits including detoxifying, anti-inflammatory, hypoglycemic, hypolipidemic, organ-system protective and anticancer effects. Among the different products generated from the plant, the essential oils are the most widely used for commercial, industrial and health promotion.

The industrial uses of the essential oils (EOs) are predominantly dependent on its insect repellent or insecticidal potentials. Studies have indicated that the essential oils extracted from selected Ocimum species are found to be efficient in killing the larvae of different mosquitoes. The EOs from *O. basilicum* has been attributed to mosquito larvicidal activity against the third instar larvae of common mosquitoes such as *Culex tritaeniorhynchus, Aedes albopictus* and *Anopheles subpictus* [5]. Similarly, the EOs from *O. gratissimum* and *O. campechianum* were shown to reduce the survival of the larval forms of *A. aegypti* and *A. albopictus* [6,7]. Further, the larvicidal potential of essential oils from O. sanctum against various mosquito species was detected [8,9]. Essential oils extracted from Ocimum plants are also efficient regulators of microbial populations as it has been reported that the essential oils from *O. tenuiflorum* [10], *O. gratissimum* [11], *O. campechianum* [12], and *O. basilicum* [13] exert antimicrobial properties against different bacterial, viral and fungal strains. Apart from these, EOs from *O. basilicum* has been effective against the *Acanthoscelides obtectus* (been weevil) and *Planococcus ficus* (grape mealybug) [14,15].

Recent developments in these aspects have indicated that the quantity of volatiles being extracted from the plant, its chemical composition percentage and the biological activities of essential oil isolated by ultrasonic assisted hydrodistillation are higher than those isolated by conventional methods [16]. However, there are no available studies on the properties of *Ocimum* essential oils isolated by ultrasound-assisted methods. Hence, the present study evaluated the chemical composition of the stress volatiles extracted, and larvicidal activity, anti-microbial properties and biological properties. Furthermore, the environmental safety of these essential oils was also evaluated using *Allium cepa* model and guppy fishes.

## 2. Results

### 2.1. Percentage Composition of Stress Volatiles in the Essential Oils by GCMS Analysis

The ultrasound-assisted hydrodistillation yielded an oil content of 2.38% (*O. gratissimum*), 2.15% (*O. basilicum*), 2.41% (*O. canum*), and 2.11% (*O. tenuiflorum*). The phytochemical profiling of the UAE essential oils extracted from different species of *Ocimum* plants was evaluated by gas chromatography-mass spectroscopy. Among the different oils, the predominant compounds are listed in Table 1 (complete list of compounds in Supplementary Table S1). In *O. canum*, thymol, linalool, camphor and p-cymene were the predominant compounds (having a % composition greater than 10%). In contrast, eugenol, camphor, and estragole were predominant in the *O. tenuiflorum* essential oil. Estragole, linalool, and methyl eugenol were predominant in the *O. basilicum*, whereas, in the *O. gratissimum*, thymol predominated.

### 2.2. In Vitro Biological Assays of Anti-Radical and Anti-Inflammatory Activity

The anti-radical activity of the *O. basilicum* essential oil was found to be higher against DPPH and hydrogen peroxide (Table 2). Likewise, the scavenging activity against ABTS radicals and FRAP capacity was higher in the *O. basilicum* and *O. gratissimum* essential oils compared to other essential oils. The inhibitory effect on inflammatory activity was estimated against lipoxygenase assays and nitric oxide production; the NO scavenging activity was high in *O. basilicum*, whereas lipoxygenase inhibition was equally high in *O. gratissimum* and *O. basilicum*.

### 2.3. Anti-Bacterial Activity

**Figure 1:**
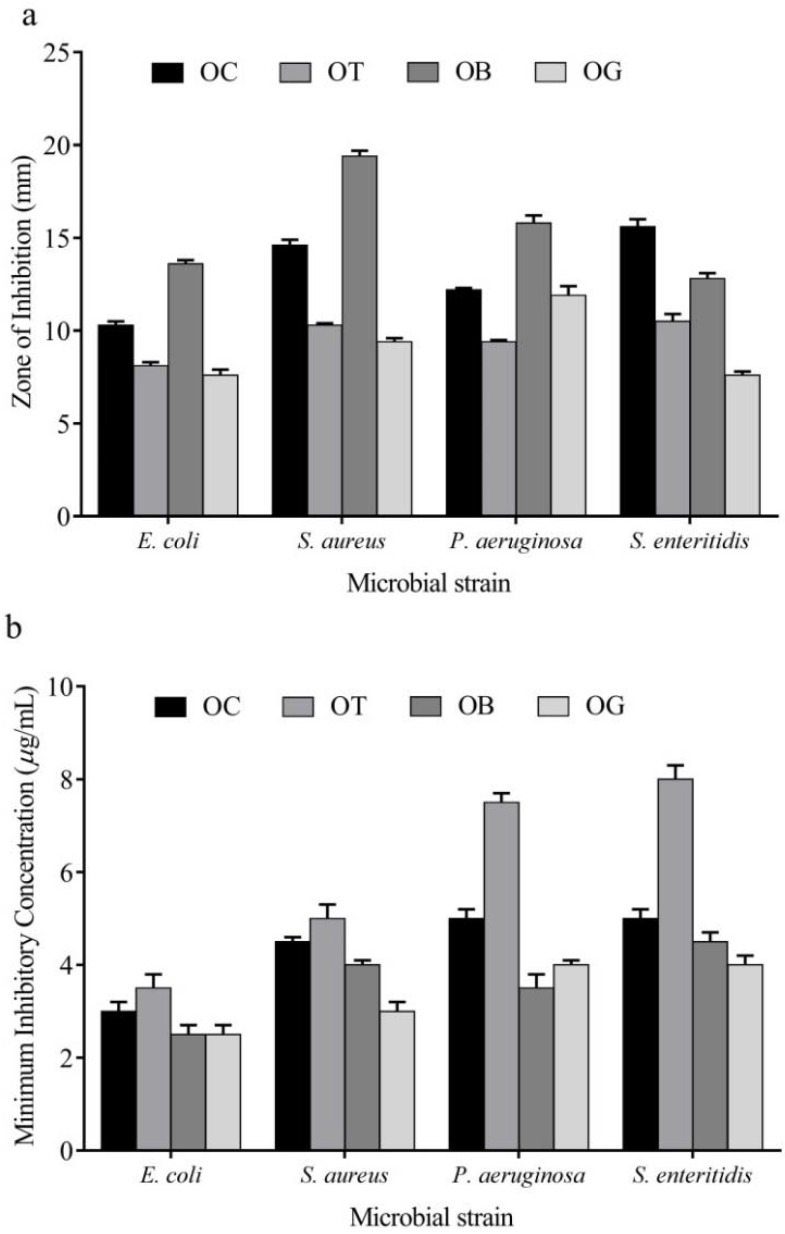
Antibacterial activity of essential oils of different species of *Ocimum* plants in terms of (**a**). zone of inhibition (mm) and (**b**) Minimum inhibitory concentrations (μg/mL).

The antimicrobial activity was estimated as antibacterial potentials; the antibacterial activity of essential oils was estimated in terms of zone of inhibition (in millimeters) in agar-well diffusion assay and also as minimum inhibitory concentration. In the disc diffusion assay, *O. gratissimum* was the most effective against *S. aureus* and *S. enteritidis* and *O. basilicum* was most effective against *P. aeruginosa* strains. Against *E. coli*, both *O. basilicum* and *O. gratissimum* essential oils were most effective (Figure 1a). Corroborating these results, the minimum inhibitory concentration against these organisms were of a similar pattern, where both *O. gratissimum* and *O. basilicum* essential oils intercepted the proliferative ability of different bacteria (Figure 1b).

### 2.4. Larvicidal Activity

**Figure 2:**
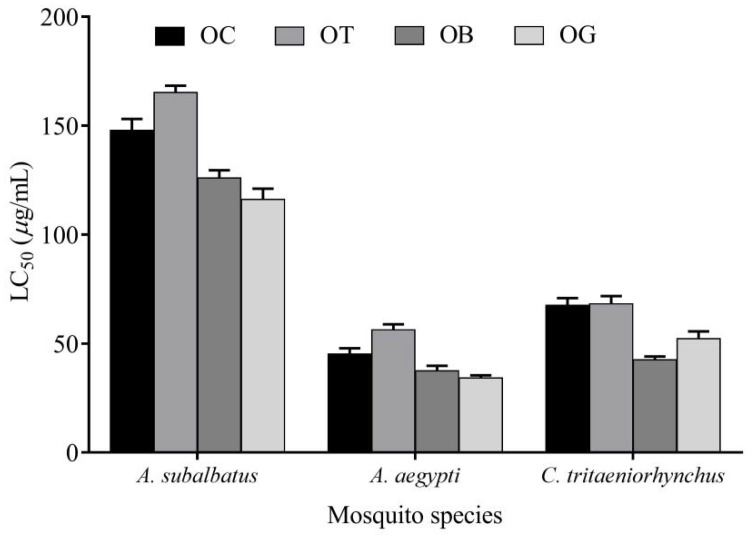
Mosquito larvicidal activity of different *Ocimum* essential oil and expressed as LC50 value in μg/mL against different species of mosquito vectors.

The *A. aegypti* was more sensitive towards *O. basilicum* and *O. gratissimum* essential oils, followed by *O. canum* and *O. tenuiflorum*. The *Culex* sp. was sensitive to *O. basilicum* essential oil. *Armigeres subalbatus* was the hardy larvae among the different mosquitoes. The sensitivity of the *A. subalbatus* was high against the essential oil of *O. gratissimum* followed by *O. basilicum* (Figure 2).

### 2.5. Toxicity on Allium Cepa Cells and Non-Targeted Organism

No significant toxicity or behavioural changes in the guppy fishes were observed until the highest concentrations tested (Supplementary Table S2). All the tested essential oils were found to have no significant behavioural changes indicative of toxicity.

## 3. Discussion

Essential oils are predominantly used in aromatherapy and cosmetic industries. Apart from that, the use of these essential oils as insecticidal compounds is emerging. Essential oils from *Ocimum* have been the focus of research in the last decade. However, previous studies used the essential oil prepared by steam or hydrodistillation. The present study analyzed the different *Ocimum* essential oils prepared by ultrasound-assisted hydrodistillation for their beneficial effects. Results indicated a significantly higher yield in the ocimum essential oil by ultrasound assisted methods; previously, it was reported that the yield of essential oil varied between 0.7–2.10% using classical hydrodistillation methods [17,18]. Hence, it is clear that ultrasound assisted hydrodistillation yields of higher quantity of stress volatiles from the selected species of *Ocimum* plants.

Apart from that, the chemical composition of the stress volatiles (essential oils) also varied from conventional methods of *Ocimum* essential oil preparation. Previous studies indicated a higher quantity of linalyl acetate and linalool in *O. basilicum* [19]; *O. gratissimum* was rich in thymol and eugenol [20]; *O. canum* contained thymol, p-cymene and γ-terpinene [21]; whereas *O. tenuiflorum* was rich in eugenol followed by β-elemene and β-caryophyllene [22]. The variation in the volatile-compound composition in this essential oil may be attributed to the ultrasound-assisted mode of extraction.

The essential oils also possessed significant antioxidant activity as indicated by the radical scavenging and reducing assays. These free radicals actively participate in the initiation events of oxidative insults to the cellular macromolecules and thereby initiation inflammatory responses in the body. Oxidative insults and subsequent chronic-inflammation are the principal conditions known to be associated with the beginning and end staged of various chronic diseases [23]. The molecules with radical scavenging/reducing potentials can therefore act as chain-breaking antioxidants and may be useful as possible nutraceuticals in the prevention of various degenerative diseases [24]. It is therefore possible that the *Ocimum* essential oil-derived molecules may emerge as strong chain-breaking antioxidants.

Furthermore, antibacterial activity was also evaluated for the *ocimum* essential oils prepared by means of ultrasound-assisted methods. Bacteria often pose a threat to the public healthcare systems and professionals due to the multiple diseases they can cause [25]. Therefore, controlling bacterial infections are of high priority in the pharmacology and biomedical research. Due to the origin of multi-drug resistance, search for novel antibiotic compounds have been hastened [26]. The different essential oils, especially from the *O. basilicum* and *O. gratissimum* were shown to have significant antibacterial properties and subsequently may emerge as significant antibacterial agents.

Mosquitoes are important vectors of various diseases; therefore, the larvicidal property is of high significance in disease prevention. The essential oils exhibited significant larvicidal properties for the *ocimum* essential oils against different species of mosquito larvae. Among these, the *Aedes aegypti* is the most common mosquito vector, which is known to transmit fevers including yellow fever, Zika, dengue, and chikungunya [27,28]. Similarly, *Culex* is also a well-known vector for many other diseases. On the contrary, the *Armigeres* species is not a common vector; however, it has been reported to transmit filarial worms and zika virus [29,30,31]. The reports have indicated that the population of *A. subalbatus* is increasing in various parts of India [32], thereby increasing the risk for various diseases.

Apart from these, the essential oils are generally considered as ecologically safe molecules; hence, they pose very limited environmental issues and health damages. The results from the non-targeted species, guppy fishes, support the assumptions. It is therefore possible that the essential oils of ultrasound-assisted methods may be employed for *Ocimum* essential-oil extraction and which exhibit significant larvicidal, antibacterial and antioxidant properties.

## 4. Materials and Methods

### 4.1. Cultivation, Collection and Extraction of Essential Oils from Ocimum Plants

The authorized and identified plant materials from the nursery were used in the study. The different species of plants including *Ocimum basilicum* (OB), *O. gratissimum* (OG), *O. tenuiflorum* (OT), and *O. canum* (OC) were cultivated in the nursery under normal atmospheric conditions with equal access to water and nutrients. The leaves were collected from these plants under a growth of 3 months. The plants were maintained in cultivation for future use.

The freshly plucked leaves of *Ocimum* plants (not old, neither tender) were cleaned with cotton, washed with fresh double distilled water and allowed to dry for 30 min in cloth. The leaves were then frozen under ultra-low temperature and grinded with glass mortar and pestle. The powdered leaf material (10 g) was then mixed with 250 mL double distilled water in a conical flask and subjected to ultrasound in a Phoenix Ultrasonic Cleaner (SGM Lab solutions, Bangaluru, India) at a frequency of 40 KHz for 30 min (input power 50 W). After 30 min, the samples were further exposed to hydrodistillation using a 300 mL capacity glass Clevenger-type apparatus (Borosil, India) for 5 h. The extracted essential oils rich in stress volatiles were dried over anhydrous sodium sulphate powder and the purified oil was kept as aliquot in small sample-storage bottles in a refrigerator (4 °C) for future use.

### 4.2. GC/MS Analysis of the Stress Volatiles of Different Ocimum Species

The instrumentation system employed for the GCMS assessment of the essential oils was Thermo Fisher Scientific, Austria; the GC measurement was started after 3 min of sample injection. Furthermore, the temperature of the chromatographic column was sustained at 40 °C for 3 min, and gradually elevated with a gradient of 8 °C and temperature of 230 °C was maintained. For the chromatography, helium was used as the carrier gas set at an automatic flow controller at a rate of 0.5 mL/min. Mass spectrometric temperature was 200 °C, with a mass range of 20–300 m/z. The possible stress volatile compounds were derived by comparing the retention indices (RI), retention time (RT), and m/z ratio by using the NIST library and comparing the literature reports.

### 4.3. In Vitro Biological Properties of Ocimum Essential Oils

Initially, the essential oil stock was aliquoted into different tubes and preserved under refrigeration; a portion of that was dissolved using 100% dimethyl sulfoxide to yield a final concentration of 25 mg/mL and using the stock, further dilutions of the drug were prepared from 0–250 µg/mL. The antioxidant property of different essential oils were identified as scavenging of the free radical generators such as DPPH, hydrogen peroxide and ABTS; in addition, the metal reducing potential of the essential oil was determined according to the methods described by Shahinuzzaman [33]. Inhibition of inflammatory enzyme 1-Lipoxygenase was conducted according to the standard protocols described in the studies of Ben-Nasr [34]. Likewise, the scavenging of Nitric oxide radicals formed were determined according the protocols described by Kakatum [35].

### 4.4. Antibacterial Activity Assay

The potential of different essential oils of *Ocimum* plants as antibacterial agents were determined by disc diffusion method [36]. The different essential oils (10 μL) were added to Whatman No.1 filter paper discs of 6 mm diameter and immersed in agarose plates containing 100 μL of different bacterial inoculum (107 CFU/mL). Methanol was used as negative control and streptomycin (20 μg/disc) as positive control. These agarose plates were kept in an incubator for 24 h at 37 °C. The experiment was repeated thrice, each conducted in duplicate and the zone of inhibition was measured in terms of diameter against each tested bacteria.

### 4.5. Determination of Minimum Inhibitory Concentration (MIC)

The determination of MIC was performed according the standard protocols described by Standard methods [37]. The bacterial cultures were maintained in the log phase of growth, and a loop of cells were transferred to lactose bile broth and incubated at 37 °C for 24 h in a bacteriological incubator. The bacterial density was set to 107 CFU/mL by spectrophotometric determination at 600 nm using appropriate dilution using fresh LB broth. Furthermore, 25 μL of the inoculum was then added to microplates containing different concentrations of ocimum essential oils (0–10 mg/mL) and incubated for 24 h. The MIC was estimated in terms of the lowest concentration of Ocimum essential oil which had no visible growth after an incubation period of 24 h.

### 4.6. Larvicidal Activity against Different Species of Mosquito Larvae

The cultures of *Armigeres subalbatus*, *Aedes aegypti*, and *Culex tritaeniorhynchus* were maintained for 10 generations. The third instar larvae (50 Nos) was collected from the colony and placed in a 50 mL beaker and various concentrations of UAE Ocimum essential oils were added by dissolving in DMSO (0.5 mL) up to 50 mL using deionized distilled water/milliQ water. 

The mortality at the end of 24 h in each concentration of essential oil was determined by counting the dead larvae (using magnifying lens on a colony counter) and percentage of death and LC50 was estimated.

### 4.7. Environmental Safety Analysis on Non-Targeted Species Toxicity in Guppy Fish (Poecilia Reticulata)

The toxicity of non-targeted organism Guppy fish (*Poecilia reticulata*) was analyzed as per the standard protocols described by Salako [38]. Guppy fishes of average body length, 3.5 ± 0.3 cm, and a weight of 1.44 ± 0.23 g were used for the study. The guppies were exposed to different concentrations of essential oils and then observed for any sign of toxicity or behavioral changes which were recorded immediately and also during 1, 6, 12, 24 and 48 h.

### 4.8. Statistical Analysis

Data of the anti-microbial studies were expressed in the standard format of Mean ± SD of four independent experiments, and each of them were conducted in triplicate. The in vitro assays were determined in three different experiments, each carried out in triplicate. The statistical meaning of comparison was drawn from a one-way ANOVA followed by Tukey–Kramer multiple comparison post hoc test.

## 5. Conclusions

Essential oils are industrially important compounds isolated from different plants or plant parts. Ultrasound-assisted hydrodistillation yielded a higher quantity of stress volatiles from different species of *Ocimum* plants with an increased yield of bioactive compounds. Further, the larvicidal, antibacterial and antioxidant activities were considerably higher in these essential oils. Among these essential oils isolated with the aid of ultrasound assisted hydrodistillation, the most active were those of *O. gratissimum* and *O. basilicum*. The study thus concludes that ultrasound-assisted hydrodistillation of *Ocimum* yields a high quality and quantity of essential oils which may have further industrial applications.

## Figures and Tables

**Table 1:** Predominant phytochemicals found in the essential oil isolated from different species of *Ocimum* (Phytochemicals present at 10% or more are included in the table. Complete chemical composition of all the essential oils are provided in Supplementary Table S1).

Compound	Retention Index	% Composition ^a^			
		*O. tenuiflorum*	*O. canum*	*O. basilicum*	*O. gratissimum*
p-Cymene	1022	1.9	12.2	3.2	15.1
Linalool	1094	3.5	21.1	25.2	7.7
Camphor	1144	26.2	13.7	7.6	10.1
Methyl Chavicol	1194	18.0	4.9	32.1	9.3
Thymol	1287	7.2	27.7	8.4	32.8
Eugenol	1355	29.0	8.4	7.2	12.3
Methyl eugenol	1401	2.1	3.7	12.1	5.5

^a^ Relative area = relative content expressed as a percentage of the total oil composition.

**Table 2:** In vitro antioxidant and anti-inflammatory activities of essential oils isolated from different species of *Ocimum* plants. The activity was estimated in terms of half-maximal inhibition concentration (IC50 value) in µg/mL.

Assay	IC_50_ Values (µg/mL)			
	*O. canum*	*O. tenuiflorum*	*O. basilicum*	*O. gratissimum*
DPPH radical scavenging	37.5 ± 2.09	52.6 ± 0.89	30.9 ± 1.32 *	44.2 ± 3.26
Hydrogen peroxide scavenging	38.2 ± 1.79	39.4 ± 2.42	27.4 ± 2.04 *	32.1 ± 2.84
ABTS radical scavenging	29.3 ± 1.52	26.3 ± 1.55	21.3 ± 2.56 *	20.3 ± 2.11 *
Ferric reducing antioxidant power	98.6 ± 3.02	121.4 ± 4.21	79.3 ± 2.77 *	93.2 ± 4.29
Nitric oxide radical scavenging	78.4 ± 4.07	94.8 ± 3.67	70.3 ± 2.33 *	80.4 ± 2.12
Lipoxygenase inhibition assay	79.3 ± 3.17	72.4 ± 2.93	60.3 ± 3.81 *	59.3 ± 2.64 *

* indicate *p* < 0.05.

## Data Availability

The data may be shared upon a valid request.

## Supplementary Materials

The following are available online. Table S1: Changes in the composition of essential oil isolated from different species of Ocimum analyzed by GC-MS; Supplementary Table S2. The indicators of toxicity in guppy fishes induced by the different Ocimum essential oils.

## Sample Availability

Samples of the compounds are available from the corresponding author. Prepared by ultrasound assisted methods (Data shown is for the highest dose 250 µg/L).

## Abbreviations

*Ocimum basilicum* (OB), *O. gratissimum* (OG), *O. tenuiflorum* (OT), *O. canum* (OC), Ultrasound-assisted hydrodistillation (UAH), Ferric reducing antioxidant power (FRAP), Nitric oxide (NO), Essential oil (EO).
