# Supplementary material for: Antimicrobial and Larvicidal Activities of Different Ocimum Essential Oils Extracted by Ultrasound-Assisted Hydrodistillation
Source: Molecules. 2022 Feb 22;27(5):1456. doi: 10.3390/molecules27051456 (PMC8911667; doi:10.3390/molecules27051456)
Supplement: Supplementary file 1 [file molecules-27-01456-s001.zip › molecules-1547600-supplementary.pdf]

Supplementary Table S1. Changes in the composition of essential oil isolated from different species of Ocimum analyzed by GC-MS

**Sl. No.**		**Retention Index**	**Compounds identified**			
			O. tenuiflorum	***O. canum***	***O. basilicum***	***O. gratissimum***
1	3-Hexen-1-ol	857	0.1	0.1	0.1	
2	α-Pinene	936	0.1			0.2
3	1-Octen-3-ol	977	0.1	1	0.1	
4	β-Pinene	977	0.2	0.1		0.9
5	3-Octanone	981	0.1		0.1	
6	Myrcene	987		0.2		
7	3-Octanol	988	0.2	0.2		0.1
8	Octanal	997	0.3	0.1	0.2	0.3
9	α-Terpinene	1016		0.2		0.1
10	p-Cymene	1022	1.9	12.2	3.2	15.1
11	Limonene	1026	0.2		0.2	0.1
12	β-Ocimene	1035	0.3	0.4		0.2
13	γ-Terpinene	1057	1.2	1.9	2	
14	Terpinolene	1085		0.4		0.2
15	Linalool	1094	3.5	21.1	24.8	7.7
16	allo-Ocimene	1130	0.6	0.5	0.1	0.8
17	Camphor	1144	26.2	13.7	7.6	10.1
18	Borneol	1167	0.1			0.2
19	Terpinen-4-ol	1174	0.2	0.2		
20	p-Cymen-8-ol	1179		0.3		0.3
21	α-Terpineol	1185	0.5		0.1	
22	Myrtenol	1193	0.5	0.2		0.2

23	Methyl Chavicol (Estragole)	1194	18	4.9	31.9	9.3
24	Nerol	1234	0.4			0.2
25	Neral	1249	0.2	0.4		0.3
26	Geraniol	1249	0.5			
27	Geranial	1264		0.2		0.3
28	Iso Bornyl acetate	1286	0.1	0.1		0.1
29	Thymol	1287	7.2	27.7	8.4	32.8
30	Carvacrol	1296	0.4	0.1		0.3
31	δ-Elemene	1385	0.9	0.1		
32	Eugenol	1355	29	8.4	7.2	12.3
33	trans-Methyl cinnamaldehyde	1375	0.2		0.2	
34	β-Elemene	1388	2.4	0.9		
35	Methyl eugenol	1401	2.1	3.7	12.1	5.5
36	α-cis-Bergamotene	1408	0.1			
37	β-Gurgunene	1430			0.1	
38	cis-β-Farnesene	1438				0.1
39	α-Humulene	1452	0.2		0.1	0.5
40	Germacrene D	1479	0.2		0.3	0.3
41	β-Selinene	1486		0.1	0.2	
42	trans-Methyl isoeugenol	1486				0.6
43	α-Selinene	1496	0.2		0.2	
44	Germacrene A	1505	0.5			0.1
45	7-Epi-α-selinene	1519		0.2	0.1	
46	δ-Cadinene	1521	0.3			
47	γ-Cadinene	1512	0.4			0.2
48	Nerolidol	1529		0.2	0.2	
49	α-Cadinene	1534		0.1		

50	Spathulenol	1576	0.1			0.1
51	1,10-di-epi-Cubenol	1668			0.1	
52	γ-Eudesmol	1631			0.1	0.2
53	epi-α-Bisabolol	1682	0.2			
54	Phytol	1939		0.1	0.3	0.3
55	Isophytol	1945	0.1			

Supplementary Table S2. The indicators of toxicity in guppy fishes induced by the different Ocimum essential oils prepared by ultrasound assisted methods (Data shown is for the highest dose 250 μg/L)

**Treatment group**	**% Mortality**	**Fishes with swimming difficulty**	**Fishes with color change**	**Time spending on top of water (Seconds)**
Normal	0	0	0	39.0 ± 5.0
O. gratissimum	0	0	0	42.0 ± 3.0
O. basilicum	0	0	0	37.0 ± 3.0
O. canum	0	0	0	40.0 ± 3.0
O. tenuiflorum	0	0	0	39.0 ± 3.0
